# Characteristics of Electrocardiogram Findings in Fulminant Myocarditis

**DOI:** 10.3390/jcdd10070280

**Published:** 2023-06-30

**Authors:** Mei-Yan Dai, Yong-Cui Yan, Lu-Yun Wang, Chun-Xia Zhao, Dao-Wen Wang, Jian-Gang Jiang

**Affiliations:** Division of Cardiology, Department of Internal Medicine, Tongji Hospital, Tongji Medical College, Huazhong University of Science and Technology, 1095 Jiefang Ave., Wuhan 430030, China; daimylch@163.com (M.-Y.D.); yongcui_yan@163.com (Y.-C.Y.); wangluyun@tjh.tjmu.edu.cn (L.-Y.W.); zhaocx@tjh.tjmu.edu.cn (C.-X.Z.); dwwang@tjh.tjmu.edu.cn (D.-W.W.)

**Keywords:** fulminant myocarditis, electrocardiogram, arrhythmias, diagnosis

## Abstract

Fulminant myocarditis (FM) is an acute and severe form of myocarditis with rapid progression and poor clinical outcomes in the absence of acute or chronic coronary artery disease. Electrocardiogram (ECG) abnormalities can provide preliminary clues for diagnosis; however, there is a lack of systemic descriptions on ECG changes in FM populations. Thus, a retrospective analysis of 150 consecutive FM patients and 300 healthy controls was performed to determine the characteristic ECG findings in FM. All patients included had markedly abnormal ECG findings. Specifically, 83 (55.33%) patients had significantly lower voltage with remarkably decreased QRS amplitudes in all leads compared with healthy controls (*p* < 0.01), and 77 (51.33%) patients had a variety of arrhythmias with lethality ventricular tachycardia/ventricular fibrillation in 21 (14.00%) patients and third-degree atrioventricular block in 21 (14.00%) patients, whereas sinus tachycardia was only found in 43 (28.67%) patients with the median heart rate (HR; 88.00 bpm, IQR: 76.00–113.50) higher than that of controls (73.00 bpm, IQR: 68.00–80.00) (*p* = 0.000). Conduction and repolarization abnormalities were common in patients. A longer QTc interval (452.00 ms, IQR: 419.00–489.50) and QRS duration (94.00 ms, IQR: 84.00–119.00) were observed in patients compared to controls (QTc interval = 399.00 ms, IQR: 386.00–414.00; QRS duration = 90.00 ms, IQR: 86.00–98.00) (*p* < 0.05). Additionally, HR > 86.50 bpm, QTc > 431.50 ms, and RV5 + SV1 < 1.715 mV can be used to predict FM. Thus, marked and severe ECG abnormalities provide preliminary clues for the diagnosis of FM.

## 1. Introduction

Acute myocarditis is a common and easily misdiagnosed disease with a high mortality rate. Confusion still exists on the proper definition and differentiation of myocarditis cause by vaccines, drugs or substances. The types of myocarditis can be classified by causative, histological and clinicopathological criteria [1]. Fulminant myocarditis (FM) is distinguished by diffuse, sudden, and serious heart inflammation, associated with myocardial edema and necrosis of the myocyte. It is a life-threatening condition, resulting in death due to cardiogenic shock, ventricular arrhythmias, or multiple organ dysfunctions [2]. The initial symptoms of FM include fever, generalized fatigue, coughing, nausea or vomiting, and headache. Particular attention should be paid to patients with both digestive and respiratory symptoms [3]. Various electrocardiogram (ECG) findings have been reported for patients with myocarditis. ECG can be easily recorded and may indicate the amount and area of damage, as indicated by cardiac magnetic resonance (CMR), which also confirms the important clinical role of ECG in the diagnosis of viral myocarditis. Patients with FM may experience abnormal ECG changes, such as ventricular tachycardia (VT), atrioventricular block (AVB), bundle branch block (BBB), ST-T changes, abnormal Q-waves, QT interval prolongation, QRS prolongation, and low voltage [3,4,5]. Nevertheless, to date, most studies have only been conducted among FM patients with a small sample size. Therefore, this study aimed to examine a group of patients with a larger sample size to elucidate the unique ECG characteristics associated with FM.

## 2. Materials and Methods

### 2.1. Study Population

We retrospectively collected data from 150 consecutive patients with FM hospitalized in Tongji Hospital, Tongji Medical College, between February 2017 and February 2023. The retrospective data collection in our study was approved by the Institutional Review Board of Tongji Hospital, Tongji Medical College, Huazhong University of Science and Technology (approval number TJ-IRB20220121) and was conducted in accordance with the principles of the Declaration of Helsinki and the International Conference on Harmonization Guidelines for Good Clinical Practice. Informed consent was obtained from all the participants or their relatives. ECGs of 300 healthy individuals from the Tongji Hospital Health Center were used as controls.

### 2.2. Diagnosis of FM

The diagnosis of FM includes both clinical and pathological diagnoses. The immediate diagnosis of FM is mostly made based on clinical manifestations. According to the Chinese expert consensus statement [6], the following requirements must be satisfied: (1) prodromal symptoms of upper respiratory or gastrointestinal viral infections, particularly extreme fatigue and poor appetite; (2) rapid development of hemodynamic compromise requiring inotropic drugs or mechanical life support (MLS); (3) serious appearance of severe heart failure symptoms (rapid decline of left ventricular ejection fraction (LVEF%) or recent onset of a conduction block) within 2 weeks with marked cardiac injury (elevation in cardiac troponin I (cTnI) and N-terminal pro-brain natriuretic peptide (NT-proBNP) levels) and cardiac hypokinesis by echocardiography and exclusion of stress cardiomyopathy and acute myocardial infarction by coronary angiography; (4) CMR- or endomyocardial biopsy (EMB)-proven myocarditis according to the Lake Louise Criteria; and (5) exclusion of other cardiac diseases, including valvular disorders, acute coronary syndrome, acute ischemic cardiomyopathy, or illnesses showing comparable clinical symptoms [6,7,8].

### 2.3. ECG Data Collection

We collected the 12-lead ECG data recorded on admission and before discharge. Our research center adopted the MedEx ECG network system for 12-lead routine ECG examination and analysis. Once ECGs were performed, data on the ECG parameters, including rhythm, heart rate, PR interval, QRS duration, QTc interval, and ST segment/T-wave changes, were obtained, analyzed automatically by the system, and reviewed by two electrophysiologists. Definitions of electrocardiography were performed according to previously published studies [9,10,11]. All ECG indices of patients with FM were compared with those of healthy controls.

### 2.4. Statistical Analysis

Data were analyzed using the Statistical Program for the Social Sciences (SPSS) version 26.0. Numerical data and categorical variables are expressed as medians (interquartile ranges (IQR)) and percentages/counts, respectively. The distributions of continuous variables between the patients and controls were compared using the Mann–Whitney U test. The Wilcoxon rank-sum test was used to examine and compare the ECGs of the patients on admission and discharge. Values of *p* < 0.05 were considered statistically significant. To further evaluate the ECG findings associated with FM, a multivariate logistic regression model was constructed with LVEF as the dependent variable. LVEF% was transformed into a categorical variable with 30% as the bound; an LVEF < 30% was considered severe systolic dysfunction [12]. A receiver operating characteristic (ROC) curve was constructed by calculating the area under the curve (AUC) to evaluate the predictive role of different parameters in FM diagnosis, and sensitivity and specificity were calculated.

## 3. Results

### 3.1. Baseline Characteristics

The baseline characteristics of the 150 patients with FM, including 76 females (50.67%) and 74 males (49.33%) with a median age of 36.33 ± 16.54 years, are summarized in Table 1. The control group included 145 (48.33%) females and 155 (51.67%) males with an average age of 38.27 ± 12.14 years. Sex and age were matched between the two groups. Among cardinal symptoms, chest distress (52.67%), fever (42.67%), fatigue (28.00%), and chest pain (22.00%) were the most common, followed by digestive and respiratory symptoms. Thirteen patients presented with syncope as the first symptom. CMR imaging was performed in 114 patients (76.00%). Coronary angiography was undertaken in 105 patients (70%), whereas EMB was only performed in 58 (38.67%) individuals. All patients received immunomodulatory therapy, antiviral therapy, and nutritional support as recommended by an expert consensus [6]. In addition to medication and general treatment, MLS was provided based on disease progression, including an intra-aortic balloon pump (76.67%), extracorporeal membrane oxygenation (24.00%), a temporary pacemaker (29.33%), continuous renal replacement therapy (30.00%), and mechanical ventilation (11.33%). The mean LVEF was 35.93 ± 14.44%, and the mean cTnI was 25639.50 ± 19327.01 pg/mL. The patients spent an average of 12.41 days at the hospital. Eight (5.33%) patients died during hospitalization or within 1 month of discharge, and the remaining patients showed significant improvement.

### 3.2. Electrocardiographic Features of FM Patients

Table 2 provides a summary of the ECG abnormalities in the patients. All ECG data revealed obvious abnormalities, with low voltage being the most common finding in 83 individuals (55.3%). Seventy-seven (51.33%) patients had various arrhythmias, and 46 and 33 patients met the diagnostic criteria for QTc prolongation and QRS widening, respectively.

Sinus tachycardia, the most common finding, was observed in 43 patients (28.67%), and 39 patients had a heart rate (HR) of 80–100 bpm. VT or VF occurred in 21 patients (14.00%) upon admission. Six (4.00%) patients presented with sinus bradycardia and another five patients required the placement of a temporary pacemaker due to high-grade AVB or sinus arrest, whereas atrial fibrillation (AF) occurred in two patients (1.33%). At discharge, three patients had permanent pacemaker implantation because of failure to recover from third-degree AVB. Five patients developed persistent AF, one of whom had AF upon admission. A total of 12 patients presented with asymptomatic sinus bradycardia partly because of the use of beta-blockers to improve long-term outcomes and 5 presented with sinus tachycardia because of incomplete recovery of cardiac function.

Conduction abnormalities were observed in 62 patients (41.33%). Third-degree AVB occurred in 21 patients (14.00%) and first-degree AVB in 4 patients (2.67%). A complete right BBB was observed in 28 patients (18.67%). Left anterior BBB occurred in five patients (3.33%), and two patients (1.33%) developed left posterior BBB. Nonspecific intraventricular block occurred in two cases (1.33%). According to the collected data, 25 patients did not recover from conduction abnormalities at discharge, including 2 cases of third-degree AVB with permanent pacemaker insertion, 16 cases of complete right BBB, 3 cases of first-degree AVB, 2 cases of left posterior BBB, and 1 case of left anterior BBB.

Repolarization abnormalities were found in 101 patients (67.33%) with 34 (22.67%) patients experiencing ST-segment elevation and 12 (8.00%) experiencing ST-segment depression. T-wave changes were the most commonly observed repolarization abnormalities occurring in 44 of 101 patients. Abnormal Q-waves were documented in 11 (7.33%) patients. On discharge, the ECG repolarization abnormalities partially recovered with 1 patient with ST-segment elevation, 3 with ST-segment depressions, and 4 with abnormal Q-waves, whereas the number of patients with nonspecific T-wave changes increased to 54.

### 3.3. ECG Comparison of FM Patients and Healthy Controls

Table 3 compares the ECG findings of the patients with those of the controls. The total QRS amplitudes in all leads showed a remarkable decrease in the patients compared to the controls (*p* < 0.01), indicating a significantly low voltage. Similarly, ST segment levels in most leads (except leads III and aVF) were significantly lower in the patients than in the controls (*p* < 0.05). Significant differences between the patients and the controls were also noted in RV5 + SV1, which also indicate the amplitude of the precordia leads. The median HR of the patients (88.00 bpm, interquartile range (IQR): 76.00–113.50) was higher than that of the controls (73.00 bpm, IQR: 68.00–80.00), i.e., 74.67% of the patients had a HR higher than that of the median of controls, respectively. Additionally, a longer QTc interval (452.00 ms, IQR: 419.00–489.50) and broader QRS width (94.00 ms, IQR: 84.00–119.00) were observed in the patients compared to the controls with a median QT interval of 399.00 ms (IQR: 386.00–414.00) and QRS wave width 90.00 ms (IQR: 86.00–98.00) (*p* < 0.05), with 85.33% and 52.67% of the patients above the median, respectively. Although some patients experienced AVB, their median PR interval, 150.00 ms (IQR: 132.00–168.00), was similar to that of the controls [median of 152.00 ms (IQR: 140.00–163.50)].

### 3.4. Comparison of ECG Findings in Patients with FM on Admission and Discharge

Table 4 compares the ECG findings of the patients on admission and discharge. After treatment, although the number of patients with low voltage decreased to 37 (24.67%) on discharge, the QRS amplitudes increased in some leads, but with no significant difference compared to that at admission, except in leads I, II, aVR, and V6. For ST-segment variations, leads II, V2, and V3 showed a significant difference between discharge and admission with the ST segment slightly depressed. Fourteen (9.33%) patients presented with QTc interval prolongation on discharge, with a median QTc interval of 430.00 ms (IQR: 401.00–455.00), which was shorter than that on admission [452.00 ms (IQR: 419.00–489.50) (*p* = 0.001)]. Ten individuals presented with QRS broadening, whereas there was no statistical difference in the QRS wave width between that at discharge and admission. The median HR of the patients with FM recovered from 88.00 bpm (IQR: 76.00–113.50) to 75.00 bpm (IQR: 67.00–87.00) on discharge.

### 3.5. Predictors of Outcome

**Figure 1:**
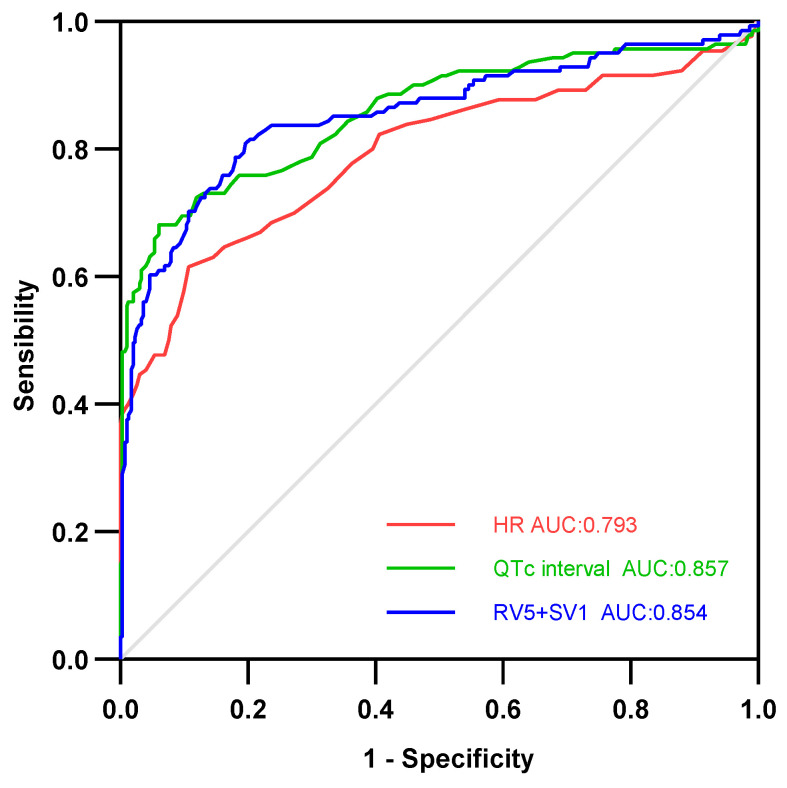
Receiver operating characteristic (ROC) curve of the ability of different ECG parameters to predict FM.

We performed a multivariate logistic regression analysis of the eligible ECG parameters selected from the univariate analysis. Multivariate logistic regression analysis revealed that the presence of sinus tachycardia [odds ratio (OR): 3.145; 95% confidence interval (CI): 1.199–8.252], low voltage [OR: 3.035; 95% CI: 1.265–7.282], QRS broadening [OR: 5.522; 95% CI: 1.781–17.120], and HR increased [OR: 1.018; 95% CI: 1.003–1.034] were significantly associated with LVEF%, and could be the predictors of heart function of FM. However, other ECG parameters, such as third-degree AVB, ventricular tachycardia or ventricular fibrillation (VT/VF), and QTc prolongation, were not significantly related to LVEF% (Table 5). Using ROC curve analysis, we evaluated the predictive role of HR, QTc interval, and RV5 + SV1 for FM. We found that HR > 86.50, (sensitivity = 0.612, specificity = 0.893, *p* < 0.0001), QTc > 431.50 (sensibility = 0.674, specificity = 0.940, *p* < 0.0001), and RV5 + SV1 < 1.72 mV (sensibility = 0.797, specificity = 0.816, *p* < 0.000) showed markedly predictive values with AUCs of 0.793, 0.857, and 0.854, respectively (Figure 1).

## 4. Discussion

To our knowledge, this is the first study to describe the ECG characteristics of a large cohort of patients with FM. The results of our study are as follows: (1) marked low-voltage of ECG was observed in all limb and precordial leads; (2) QTc was prolonged and QRS broadened significantly in 30.67% of patients meeting the criteria for QTc prolongation and 22.00% of patients having a QRS duration of ≥120 ms; (3) lethal arrhythmias occurred in approximately 30% patients, including those with sinus arrest or third-degree AVB, VT, or VF; (4) interestingly, the median rate of sinus rhythm was 88.00 bpm (IQR: 76.00–113.50), which was significantly higher than that of the controls [73.00 bpm (IQR: 68.00–80.00)]. Further, only 28.67% patients had sinus tachycardia, but 74.67% of the patients had a HR higher than that of the median controls, respectively. These findings demonstrate that patients with FM display marked and severe ECG abnormalities, which indicate a loss of myocardial electrical activity and injury during conduction, although most of these are temporary. These ECG abnormalities are not specific but differ from those without FM; most importantly, they provide the preliminary clues for the diagnosis of FM.

A previous study found that clinically significant arrhythmias are associated with low ECG voltages and worse clinical outcomes in acute myocarditis [13]. Sinus tachycardia is typical of FM and reflects the degree of systemic inflammation and/or hemodynamic impairment [14]. However, the proportion of patients with sinus tachycardia was lower than what was expected in our study, with 74.67% of the patients having a HR higher than that of the median controls. The resting HR is regarded as a marker of autonomic nervous system activity that demonstrates sympathetic hyperactivity when elevated [15]. When the HR increases, it can put individuals in danger of arrhythmias and lead to higher cyclic stretch and elastin fatigue [16]. Inflammatory markers such as CRP or IL-6 have been confirmed to influence the HR by affecting the autonomic nervous system [17,18]. In addition, a “cytokine storm” was proposed to play an important role in the pathophysiology of FM based on the plasma cytokine profile [7]. These findings suggest that inflammation plays an important role in the development of arrhythmia. QTc interval prolongation has also been proposed as an established potential arrhythmogenic trigger and risk factor for ventricular arrhythmia and death [19,20]. Furthermore, myocardial interstitial edema caused by inflammation and direct myocardial injury (apoptosis and necrosis) can result in ventricular wall thickening and impaired ventricular contractility, which can exacerbate heart arrhythmias [13,21,22,23]. We identified a high incidence of serious arrhythmias in patients with FM, including those with sinus arrest, third-degree AVB, VT, or VF, which is consistent with the previously published literature [24,25], but we did not demonstrate that these were independent predictors for FM in this study, except for sinus tachycardia, low voltage, QRS broadening, and HR, partly due to the early application of comprehensive treatment programs based on life support, which reduces the load of the heart and resolves myocardial inflammation and edema. Based on these findings, HR reduction is recommended in the management of patients with FM [26].

As underlined by 2013 ESC Task Force, QRS complex alterations in acute myocarditis include low voltages, abnormal Q waves, and intraventricular conduction delay/BBB [27], which were confirmed in our study. Widespread low QRS voltage is a possible ECG manifestation of myopericarditis with pericardial effusion resulting from increased resistance from the accumulated fluid. However, regardless of whether it is accompanied by pericardial effusion, the amplitude of the QRS wave in the ECG of patients with FM is significantly lower than that in the recovery period or normal controls, and its pathogenesis may be related to myocardial inflammation edema, pulmonary edema, and peripheral tissue edema [24,28,29,30]. In a retrospective study of patients with myocarditis, an acute fulminant course was predicted by a prolonged QRS complex [31]. An abnormal QRS complex was linked to worse survival and lower LV function in a small study of biopsy-proven myocarditis [32]. In addition, prolonged QRS duration is an independent predictor of cardiac death or heart transplantation in patients admitted to the hospital with suspected myocarditis without previous heart failure [11]. Similar to the QT interval prolongation, a wide QRS duration may result from a disruption in cardiomyocyte integrity caused by the disconnection of actin-based cytoskeletal and sarcomeric structures from membrane-bound dystrophin-associated glycoproteins and external basement membrane [33]. These disruptions induce changes in the cardiomyocyte membrane potential, resulting in myocardial conduction system dysfunction [34]. We confirmed that lower LVEF% was significantly associated with sinus tachycardia, low voltage, QRS broadening (including LBBB, RBBB, and intraventricular block), and HR in patients with FM. Pathological Q waves are typical of acute myocardial infarction (AMI), but they can also be present in FM patients with the same characteristics that manifest as acute coronary syndrome (ACS), such as myocarditis. Previous studies have shown that the presence of pathological Q waves is associated with poorer prognosis in FM patients, especially in those with ST-segment elevation [35]. Unlike AMI, myocarditis should be highly suspected in patients with extensive lead ST-segment elevation without reciprocal ST-segment depression or when ECG changes cannot be explained by coronary angiography findings [36]. Clinical factors supporting a diagnosis of myocarditis include lower patient age and complaints of recent viral illness; however, these symptoms are nonspecific, and show slowly evolving ECG changes involving more than one vascular territory and diffuse or absent (rather than focal) wall motion abnormalities on ECG [37]. The early disappearance of pathological Q waves and ST segment reduction suggests reversible myocardial injury and resolution of inflammatory processes in patients with FM. These results are consistent with the present findings, which are based on a larger cohort of patients.

We demonstrated a high prevalence (67.33%) of repolarization abnormalities in patients with FM, which is in line with a previous study [36]. Notably, ST-T changes in myocarditis patients evolve as the disease progresses. Certain patients with ST-segment elevation or depression return to normal over several days, whereas others progress further to T-wave inversion [5]. ST segment changes in FM may reflect ongoing myocardial edema that leads to the worsening of cell membrane leakage, accumulation of bioproducts, and a decrease in energy delivery and oxygenation of the myocardial tissue [13]. ST depression was less frequent in the FM patients in our study; however, it was not specific and did not show a reciprocal change in ST segment elevation, which is representative of one of the earliest clinical signs of FM [14,38]. Although some prognostic values have been previously described [39], in the present study, neither T-wave inversion nor ST-segment depression was associated with cardiac function, which is consistent with a previous study [36].

This study described the ECG characteristics of patients with FM in detail. However, this study had several limitations. First, the ECG data of some patients were incomplete owing to the retrospective nature of the study, and ECG changes must be reviewed dynamically. The small amount of missing data did not affect the final conclusion of our study. Second, because the first ECG was performed on hospital admission, the true time of onset of ECG changes, particularly QRS prolongation and initial arrhythmias, could not be determined. However, the data reflected the QRS duration at the onset of symptoms. The use of drugs that prolong the QT interval may have influenced the results. Lastly, the factors influencing the prognosis of patients with FM were not investigated.

## 5. Conclusions

Patients with FM showed a variety of dynamic changes on ECG, showing sinus tachycardia, low voltage, ST-segment depression, QTc and QRS prolongation, and lethal arrhythmias as significant characteristics. The HR, QTc prolongation, and RV5 + SV1 were independent predictors of FM. Although some ECG changes are temporary, these readily available ECG parameters provide important information for the management of patients with suspected FM.

## Figures and Tables

**Table 1:** Baseline characteristics of the patients with FM and the controls.

Characteristic	FM N = 150	Control N = 300
Age (year)	36.33 ± 16.54	38.27 ± 12.14
Male	74 (49.33%)	155 (51.67%)
Cardinal symptoms		
Chest distress	79 (52.67%)	
Fever	64 (42.67%)	
Fatigue	42 (28.00%)	
Chest pain	33 (22.00%)	
Palpitation	22 (14.67%)	
Dyspnea	21 (14.00%)	
Diarrhea	14 (9.33%)	
Syncope	13 (8.67%)	
Vomiting	11 (7.33%)	
Dizzy	11 (7.33%)	
Cough	9 (6.00%)	
Abdominal pain	5 (3.33%)	
Cardiac MRI performed	114 (76.00%)	
Coronary angiography	105 (70.00%)	
Endomyocardial biopsy	58 (38.67%)	
Mechanical life support		
IABP	115 (76.67%)	
CRRT	45 (30.00%)	
Temporary pacemaker	44 (29.33%)	
ECMO	36 (24.00%)	
Invasive mechanical ventilation	17 (11.33%)	
LVEF%	35.93 ± 14.44	
cTnI (pg/mL)	25,639.50 ± 19,327.01	
Hospitalization stay (day)	12.41 ± 6.45	
Outcome of death	8 (5.33%)	

MRI, magnetic resonance imaging; IABP, intra-aortic balloon; CRRT, continuous renal replacement therapy; ECMO, extracorporeal membrane oxygenation; LVEF, left ventricular ejection fraction. Data showed with mean ± SD for age, LVEF%, cTnI and hospitalization stay, with numbers and percentages for the other data.

**Table 2:** ECG abnormalities in patients with FM.

Characteristic	Admission *n* (%)	Discharge *n* (%)
Low voltage	83 (55.33%)	37 (24.67%)
QTc prolongation	46 (30.67%)	14 (9.33%)
QRS broadening	33 (22.00%)	10 (6.67%)
Abnormal Q wave	11 (7.33%)	4 (2.67%)
Origin abnormalities		
Sinus tachycardia	43 (28.67%)	5 (3.33%)
VT/VF	21 (14.00%)	0 (0.00%)
Escape rhythm	14 (9.33%)	2 (1.33%)
Sinus bradycardia	6 (4.00%)	12 (8.00%)
Pacemaker rhythm	5 (3.33%)	3 (2.00%)
Atrial fibrillation	2 (1.33%)	5 (3.33%)
Conduction abnormalities		
Rright bundle branch block	28 (18.67%)	16 (10.67%)
Third-degree AVB	21 (14.00%)	2 (1.33%)
Left anterior bundle branch block	5 (3.33%)	2 (1.33%)
First-degree AVB	4 (2.67%)	3 (2.00%)
Left posterior bundle branch block	2 (1.33%)	2 (1.33%)
Nonspecific intraventricular block	2 (1.33%)	0 (0.00%)
ST-T changes		
T wave changes	44 (29.33%)	54 (36.00%)
ST segment elevation	34 (22.67%)	1 (0.67%)
ST segment depression	12 (8.00%)	3 (2.00%)

VT/VF, ventricular tachycardia/ventricular fibrillation; AVB, atrioventricular block.

**Table 3:** Comparison of the ECG findings between FM patients and controls.

Characteristic	Median (Interquartile Range)		*p* Value	*n* (%) ^c^
	FM	Control		
QRS amplitudes (mV)				
II	0.60 (0.37–0.86)	1.07 (0.86–1.32)	0.000	122 (81.33%)
III	0.50 (0.32–0.82)	0.67 (0.45–0.96)	0.001	95 (63.33%)
aVF	0.49 (0.30–0.79)	0.83 (0.60–1.07)	0.000	109 (72.67%)
I	0.40 (0.28–0.55)	0.61 (0.43–0.80)	0.000	112 (74.67%)
aVL	0.33 (0.22–0.55)	0.43 (0.29–0.63)	0.003	92 (61.33%)
aVR	0.39 (0.27–0.56)	0.77 (0.65–0.92)	0.000	131 (87.33%)
Sum of Limb leads	2.83 (2.04–3.98)	4.50 (3.66–5.29)	0.000	117 (78.00%)
V1	0.65 (0.38–0.93)	1.06 (0.77–1.35)	0.000	115 (76.67%)
V2	1.17 (0.66–1.70)	1.87 (1.53–2.41)	0.000	115 (76.67%)
V3	1.19 (0.76–1.72)	1.92 (1.53–2.43)	0.000	119 (79.33%)
V4	1.15 (0.77–1.78)	1.97 (1.57–2.50)	0.000	117 (78.00%)
V5	0.96 (0.64–1.44)	1.66 (1.40–2.03)	0.000	116 (77.33%)
V6	0.75 (0.42–1.01)	1.28 (1.06–1.55)	0.000	122 (81.33%)
Sum of chest leads	5.98 (4.03–8.27)	9.91 (8.38–11.87)	0.000	120 (80.00%)
ST segment change (mV)				
II	0.03 (−0.02–0.10)	0.05 (0.02–0.08)	0.009	81 (54.00%)
III	0.01 (−0.02–0.06)	0.00 (−0.01–0.03)	0.296	63 (42.00%)
aVF	0.02 (−0.02–0.08)	0.03 (0.01–0.06)	0.053	81 (54.00%)
I	0.02 (−0.01–0.07)	0.04 (0.02–0.07)	0.000	93 (62.00%)
aVL	0.00(−0.03–0.05)	0.02 (0.00–0.03)	0.002	85 (56.67%)
aVR	−0.02 (−0.07–0.01)	−0.04 (−0.07–−0.02)	0.000	82 (54.67%)
V1	0.03 (−0.01–0.12)	0.05 (0.02–0.09)	0.002	79 (52.67%)
V2	0.11 (0.04–0.27)	0.19 (0.12–0.29)	0.000	89 (59.33%)
V3	0.11 (0.03–0.26)	0.17 (0.09–0.25)	0.004	80 (53.33%)
V4	0.06 (0.00–0.18)	0.12 (0.06–0.19)	0.000	85 (56.67%)
V5	0.02 (−0.03–0.12)	0.08 (0.04–0.13)	0.000	88 (58.67%)
V6	0.01 (−0.04–0.09)	0.05 (0.02–0.09)	0.000	85 (56.67%)
Other indicators				
Heart rate (bpm)	88.00 (76.00–113.50)	73.00 (68.00–80.00)	0.000	112 (74.67%)
P wave width (ms)	92.00 (76.00–100.00)	100.00 (92.00–104.00)	0.000	99 (66.00%)
PR interval (ms) ^a^	150.00 (132.00–168.00)	152.00 (140.00–163.50)	0.869	67 (44.67%)
QRS wave width (ms) ^b^	94.00 (84.00–119.00)	90.00 (86.00–98.00)	0.023	79 (52.67%)
QTc interval (ms)	452.00 (419.00–489.50)	399.00 (386.00–414.00)	0.000	128 (85.33%)
RV5 + SV1	1.03 (0.46–1.60)	2.15 (1.81–2.59)	0.000	124 (82.67%)

^a^ Patients with atrial fibrillation/flutter were excluded. ^b^ Patients with cardiac pacemakers were excluded. ^c^ The number and proportion of patients with higher-than-normal values.

**Table 4:** Comparison of the ECG findings of FM patients at admission and discharge.

Characteristic	Median (Interquartile Range)		*p* Value
	Admission	Discharge	
QRS amplitudes (mV)			
II	0.60 (0.37–0.86)	0.69 (0.43–0.98)	0.017
III	0.50 (0.32–0.82)	0.48 (0.31–0.72)	0.706
aVF	0.49 (0.30–0.79)	0.53 (0.30–0.80)	0.671
I	0.40 (0.28–0.55)	0.50 (0.37–0.80)	0.000
aVL	0.33 (0.22–0.55)	0.37 (0.26–0.51)	0.556
aVR	0.39 (0.27–0.56)	0.59 (0.42–0.75)	0.000
V1	0.65 (0.38–0.93)	0.81 (0.52–1.07)	0.111
V2	1.17 (0.66–1.70)	1.27 (0.84–1.86)	0.062
V3	1.19 (0.76–1.72)	1.29 (0.83–1.82)	0.366
V4	1.15 (0.77–1.78)	1.25 (0.85–1.95)	0.418
V5	0.96 (0.64–1.44)	1.07 (0.71–1.50)	0.176
V6	0.75 (0.42–1.01)	0.85 (0.52–1.20)	0.010
ST segment change (mV)			
II	0.03 (−0.02–0.10)	0.02 (0.00–0.05)	0.045
III	0.01 (−0.02–0.06)	0.00 (−0.02–0.03)	0.641
aVF	0.02 (−0.02–0.08)	0.01 (0.00–0.03)	0.411
I	0.02 (−0.01–0.07)	0.02 (0.00–0.05)	0.256
aVL	0.00 (−0.03–0.05)	0.01 (0.00–0.04)	0.694
aVR	−0.02 (−0.07–0.01)	−0.02 (−0.05–0.00)	0.630
V1	0.03 (−0.01–0.12)	0.03 (0.00–0.06)	0.336
V2	0.11 (0.04–0.27)	0.08 (0.03–0.14)	0.012
V3	0.11 (0.03–0.26)	0.08 (0.02–0.14)	0.003
V4	0.06 (0.00–0.18)	0.05 (0.00–0.10)	0.050
V5	0.02 (−0.03–0.12)	0.03 (−0.01–0.08)	0.176
V6	0.01 (−0.04–0.09)	0.01 (−0.01–0.05)	0.736
Other indicators			
Heart rate (bpm)	88.00 (76.00–113.50)	75.00 (67.00–87.00)	0.000
P wave width (ms)	92.00 (76.00–100.00)	92.00 (81.00–101.50)	0.320
PR interval (ms) ^a^	150.00 (132.00–168.00)	154.00 (134.00–171.50)	0.537
QRS wave width (ms) ^b^	94.00 (84.00–119.00)	92.00 (84.00–103.50)	0.116
QTc interval (ms)	452.00 (419.00–489.50)	430.00 (401.00–455.00)	0.000
RV5 + SV1	1.03 (0.46–1.60)	1.39 (0.90–1.91)	0.002

^a^ Patients with atrial fibrillation/flutter were excluded. ^b^ Patients with cardiac pacemakers were excluded.

**Table 5:** Multivariate regression analysis of the ECG characteristics associated with LVEF% of FM.

Variable	OR (95% CI)	*p*-Value
Sinus tachycardia	3.145 (1.199–8.252)	0.020
Third-degree AVB	0.668 (0.158–2.817)	0.582
VT/VF	2.056 (0.649–6.517)	0.221
Low voltage	3.035 (1.265–7.282)	0.013
QTc prolongation	1.548 (0.623–3.847)	0.347
QRS broadening	5.522 (1.781–17.120)	0.003
Heart rate	1.018 (1.003–1.034)	0.021

OR, odds ratio; CI, confidence interval.

## Data Availability

The data that support the findings of this study are available on request from the corresponding author upon reasonable request.
